# Hydrophobic Effect of Amphiphilic Derivatives of Chitosan on the Antifungal Activity against *Aspergillus flavus* and *Aspergillus parasiticus*

**DOI:** 10.3390/molecules18044437

**Published:** 2013-04-15

**Authors:** Ricchard Hallan Felix Viegas de Souza, Mirelli Takaki, Rafael de Oliveira Pedro, Juliana dos Santos Gabriel, Marcio José Tiera, Vera Ap. de Oliveira Tiera

**Affiliations:** Department of Chemistry and Environmental Sciences, Institute of Biosciences, Humanities and Exact Sciences–IBILCE, São Paulo State University–UNESP, São José do Rio Preto, São Paulo, 15054-000, Brazil

**Keywords:** chitosan, antifungal activity, derivatives, amphiphilic, *Aspergillus flavus*, *Aspergillus parasiticus*

## Abstract

Low molecular weight amphiphilic derivatives of chitosan were synthesized, characterized and their antifungal activities against *Aspergillus flavus* and *Aspergillus parasiticus* were tested. The derivatives were synthesized using as starting material a deacetylated chitosan sample in a two step process: the reaction with propyltrimethyl-ammonium bromide (Pr), followed by reductive amination with dodecyl aldehyde. Aiming to evaluate the effect of the hydrophobic modification of the derivatives on the antifungal activity against the pathogens, the degree of substitution (DS_1_) by Pr groups was kept constant and the proportion of dodecyl (Dod) groups was varied from 7 to 29% (DS_2_). The derivatives were characterized by ^1^H-NMR and FTIR and their antifungal activities against the pathogens were tested by the radial growth of the colony and minimum inhibitory concentration (MIC) methods. The derivatives substituted with only Pr groups exhibited modest inhibition against *A. flavus* and *A. parasiticus*, like that obtained with deacetylated chitosan. Results revealed that the amphiphilic derivatives grafted with Dod groups exhibited increasing inhibition indexes, depending on polymer concentration and hydrophobic content. At 0.6 g/L, all amphiphilic derivatives having from 7.0 to 29% of Dod groups completely inhibited fungal growth and the MIC values were found to decrease from 4.0 g/L for deacetylated chitosan to 0.25–0.50 g/L for the derivatives. These new derivatives open up the possibility of new applications and avenues to develop effective biofungicides based on chitosan.

## 1. Introduction

Over the past decades, chitosan, due to its unique properties such as its natural antimicrobial activity, biocompatibility and biodegradability, has received significant attention for the development of new biofungicides [1,2]. These properties, combined with its structure which is particularly susceptible to react through nucleophilic attack, makes chitosan an excellent source for this purpose [3]. In this regard, a variety of chemical modifications targeting the free amine and/or the hydroxyl groups of chitosan have been employed to obtain antimicrobial materials [4]. However, several studies have shown that its antimicrobial activity may depend on various intrinsic factors, such as, the type of pathogen, the molecular weight (Mw), the positive charge density and hydrophilic/hydrophobic characteristics [5]. The positive charge density is one of the most important parameters and depends on the degree of deacetylation (DD). The protonation of the amino groups of deacetylated chitosan provides positive charges to the macromolecular chain, which in turn, allows the interaction with the negatively charged microbial cell membranes. For instance, it has been shown that the antimicrobial activity of oligochitosans may vary greatly depending on the type of pathogen, and at neutral pH, the deprotonation of the amino groups reduced the inhibition of the mycelial growth against *P. capsici* [6] from 86% to 28%.

Aiming to improve the properties of chitosan, the modification of its structure has been widely investigated, not only to increase the antimicrobial activity, but also to obtain derivatives soluble over a wide range of pH values. Chitosan has been chemically modified to produce quaternary ammonium salts in order to improve its antimicrobial activity against *Listeria monocytogenes* and *Salmonella typhimurium*. The trimethylated chitosan exhibited superior antibacterial activity compared to chitosan and its higher activity was attributed to the permanent positive charges on the chitosan chain [7]. Moreover, the importance of cationic charges has been reinforced by comparing antifungal activity of chitosan with carboxymethyl chitosan on *Candida albicans* [8], and other pathogens [9], demonstrating that the presence of carboxylic groups decreases the polycationic character, affecting the interaction with the microbial cell surface. The antifungal activity has also been shown to depend on the molecular weight of the chitosan [10], and of the hydrophobicity of the derivatives [11,12]. The exact mechanism is still not clear, but the activity is partially credited to the electrostatic attraction between the polycations and negatively charged cells walls [5]. However, it has been shown that, positively charged chains are important, but not sufficient to provide an efficient activity, and that, depending on the type of pathogen, oligomers of chitosans may exhibit antimicrobial activities either lower [13] or higher [4,6] than that of high molecular weight chitosan. Regarding the hydrophobicity of the derivatives, recent results indicate that hydrophobic groups linked to chitosan chain may increase the antimicrobial activity [11], but for derivatives having high degrees of substitution the activity can decrease again [12]. Moreover, it has been recently reported that the antimicrobial activity of amphiphilic derivatives depends on the molecular weight, and that lipophilic chitooligasaccharides exhibited higher activity than those more hydrophilics [4]. On the contrary, quaternary lipophilic derivatives based on the chitosan polymer exhibited lower antibacterial activity than their more hydrophilic counterparts [4]. 

The fungi *Aspergillus flavus* and *Aspergillus parasiticus* are commonly found in tropical and subtropical climates and are considered a threat to the production of several oilseed crops, due to the production of mycotoxins, such as aflatoxins, which are carcinogenic and may cause substantial economic losses [14]. We have recently reported the synthesis and characterization of new chitosan derivatives synthesized by the reaction of deacetylated chitosan (CH) with propyl and pentyltrimethylammonium bromides to obtain derivatives with increasing degrees of substitution. We showed that the antifungal activity of chitosan against *Aspergillus flavus* is improved by increasing the degree of substitution of alkytrimethylammonium groups on the polymer chain. Moreover, the results indicated that the hydrophobicity of the derivatives play an important role on the antifungal activity against this fungus. 

**Scheme 1:**
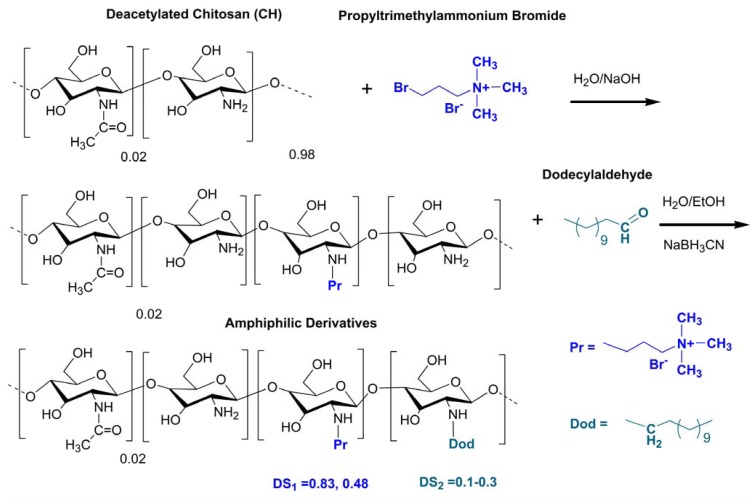
Schematic representation of the synthesis of the amphiphilic derivatives of chitosan.

In this work we extended the previous findings by applying new amphiphilic derivatives of chitosan against *A. flavus* and *A. parasiticus*. The synthesis procedure was carried out in two steps and the hydrophocity was varied by increasing the degree of substitution by Dod groups (DS_2_) attached to the backbone of the quarternary derivatives (Scheme 1). The antifungal activities of the chitosan derivatives against the fungi *A. flavus* and *A. parasiticus* are described and discussed, taking into account the degree of substitution of the substituted derivatives and their hydrophobicities.

## 2. Results and Discussion

### 2.1. Synthesis and Characterization of the Amphiphilic Derivatives

**Figure 1:**
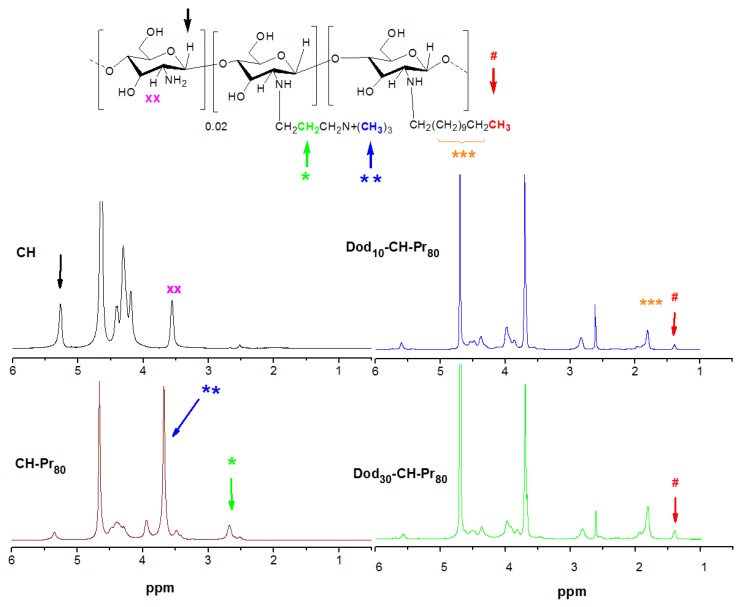
^1^H-NMR spectra of deacetylated chitosan (**CH**), quaternary derivative (**CH-Pr_80_**) and its amphiphilic derivatives (**Dod_30_-CH-Pr80**). The resonance of the methylene protons (green color) were used to determine DS_1_ and the areas corresponding to methyl protons (red color) of Dod groups were used to determine DS_2_; temperature: 70 °C; solvent: D_2_O/DCl (1%).

We have previously shown that chitosan derivatives containing increasing amounts of quaternary amino groups may be synthesized by a simple and reliable method using bromo- propyltrimethylammonium bromides [15]. The degree of substitution by Pr groups may be reasonably well controlled by setting the initial molar ratio of the bromoalkyltrimethylammonium species to glucosamine units. The amphiphilic derivatives were obtained by a further modification using the reductive amination reaction with dodecylaldehyde. The ^1^H-NMR of deacetylated chitosan and its corresponding quarternary and amphiphilic derivatives are shown in Figure 1. The degree of deacetylation of chitosan was 98.5 mol % of glucosamine units, and was determined from the ^1^H-NMR spectrum using the areas of the peaks at δ 2.51 ppm and δ 3.6 ppm, which correspond to the acetamido methyl protons and the proton linked to carbon 2 of the gluocosamine ring, respectively [16]. The success of the synthesis of the amphiphilic derivatives was confirmed by the appearance of characteristic peaks of both groups, the propyltrimethylammonium (Pr) and dodecyl groups (Dod). The ^1^H-NMR spectrum of both quaternary derivatives denoted as CH-Pr_80_ and CH-Pr_50_ exhibited peaks at δ 2.6 ppm and δ 3.7 ppm, corresponding to the resonances of the methylene protons [(CH_3_)_3_N^+^-CH_2_C***H*_2_**-CH_3_] and methyl protons [-CH_2_CH_2_CH_2_N^+^(***CH_3_***)_3_] of the Pr groups. The degrees of substitution by the quaternary groups were determined as described in Section 3.3.2 using the Equation 1, and the values obtained were 83.0 and 48.0% for CH-Pr_80_ and CH-Pr_50_, respectively. The presence of Dod groups was confirmed by the appearance of peaks at δ 1.45 ppm and 1.7–2.0 ppm, due to the resonance of methyl protons and methylene protons of the hydrocarbon chain, respectively (Figure 1). The areas of the peak at 1.45 ppm were used to determine the degrees of substitution by Dod using Equation (2). The molecular weight was determined by viscosity measurements and, as can be seen from Table 1, the molecular weight (Mv) decreased about 2.5-fold, which probably occurred due to the alkaline medium and the long reaction time.

### 2.2. Antifungal Activity of Deacetylated Chitosan and Its Amphiphilic Quaternary Derivatives

**Figure 2:**
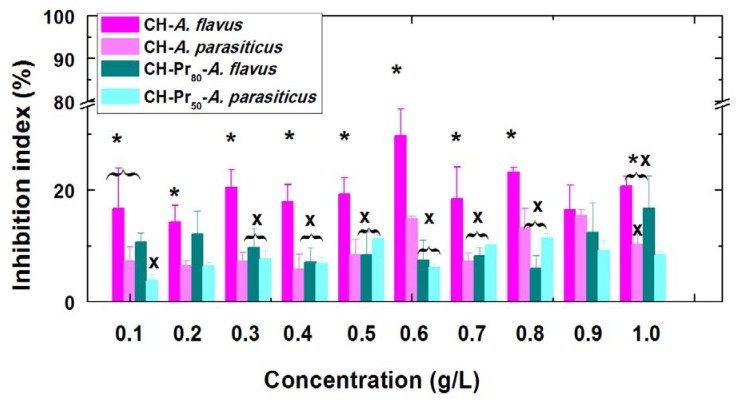
Concentration effect on the inhibition index of deacetylated chitosacn (CH) and its quartenary derivatives (CH-Pr80 and CH-Pr50) against *A. flavus* and *A. parasiticus.* Bars within the same concentration with different symbols are significantly different according to Kruskal-Wallis test (*p <* 0.05).

Several authors have shown that the presence of permanent cationic charges on the chitosan backbone improves the antifungal activity against various microorganisms [17,18]. This trend was previously confirmed by our research group working on *Aspergillus flavus*, and may be due to strengthening of the interaction between the polycation and cell wall of the fungus [15]. In this work the main goal was to evaluate the effect of the hydrophobic modification of quaternary derivatives on the antifungal activity against *A. flavus* and *A. parasiticus*. For that, the concentration of deacetylated chitosan (CH) and its amphiphilic derivatives was varied from 0.1 to 1.0 g/L and the inhibition percentages were calculated as described in the Experimental (Section 3.5). It has been shown that CH exhibits a modest inhibition (5–15%) against *A. flavus* [15] and no clear trend was observed in the polymer concentration range studied (Figure 2). Similar results were also obtained with *A. parasiticus*, however, the inhibition percentages against *A. parasiticus* was smaller over all concentration range, on average around 10%. As can be seen in Figure 2 the inhibition indexes obtained against *A. parasiticus* were statistically different (with *p* < 0.05) of those obtained with *A. flavus*, over the concentration range of 0.1 to 0.8 g/L.

**Table 1:** Properties of chitosan and its amphiphilic derivatives used for antifungal studies.

Derivative	Dod/NH_2_ ratio	[*η*] (mL/g)	Mv (g/mol) ×10^3^	DS_2_(%) ** Dod groups
CH	-	267.7	27.2	
CH-Pr_80_^*^	-	101.3	8.1	-
Dod_10_-CH-Pr_80_	0.10			8.3
Dod_15_-CH-Pr_80_	0.15			14.1
Dod_30_-CH-Pr_80_	0.30			29.0
CH-Pr_50_^*^	-	142.6	12.3	-
Dod_5_-CH-Pr_50_	0.10			7.1
Dod_15_-CH-Pr_50_	0.15			9.4
Dod_30_-CH-Pr_50_	0.30			27.1

Degree of deacetylation (DD) = 98.2% by ^1^H-NMR; * DS_1_ Determined by ^1^H-NMR measurements using Equation 1 was 83.0% and 48.0%, respectively. ** Determined by ^1^H-NMR measurements using Equation 2 (section, 3.3.2).

Recent reports in the literature show that chitosans having higher degrees of deacetylation are more effective in inhibiting the antimicrobial grown of several pathogens [5] and the inhibition indexes reported in this work for CH against *A. parasiticus*, are similar to those recently obtained by Cota-Arriola *et al.*, whose results showed that at concentrations of 3.0 and 4.5 g.L^−1^ fungistatic inhibition reached the highest values [19]. The fungistatic activity of deacetylated chitosan against *A. flavus* has been also confirmed by several authors using films of chitosan and its derivatives [20,21].

However, as can be seen from Figure 2, the inhibition indexes of both quaternary derivatives, CH-Pr_8__0_ and CH-Pr_50_ against *A. flavus* and *A. parasiticus* remained around 8-10%, which were lower than those obtained using CH. Although unexpected, this result may be due to the lower molecular weights of the quaternary derivatives compared to CH. At pH 5.0, essentially all free amino groups of CH are protonated and therefore similarly to the quaternary derivatives, the macromolecular chains possess a high positive charge density. However, the quaternary derivatives were soluble over a wide range of pH, and are more hydrophilic than CH, accordingly, it can be postulated that these derivatives interact less efficiently with the fungi cell walls than CH does, resulting in the slightly smaller inhibition indexes for the quaternary derivatives. 

**Figure 3:**
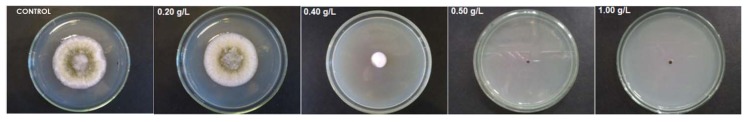
Effect of increasing concentrations of Dod_30_-CH-Pr_80_ on the radial growth of *A. flavus* after the 7^th^ day of inoculation. From left to right: Control, 0.2; 0.4, 0.5 and 1.0 g/L.

On the contrary, the attachment of Dod groups to CH-Pr_80_ and CH-Pr_50_ abruptly increased the antifungal activity compared to both CH and the quaternary derivatives. The addition of amphiphilic quaternized chitosan derivatives to the BDA medium significantly inhibited the mycelial growth of both fungi as the concentration was increased. A representative experiment comparing the effect of increasing concentrations of Dod_30_-CH-Pr_80_ on the radial growth of *A. flavus*, after the 7^th^ day of inoculation is shown in Figure 3 and, as can be seen at 0.5 g.L^−1^, the fungal growth was completely inhibited. 

**Figure 4:**
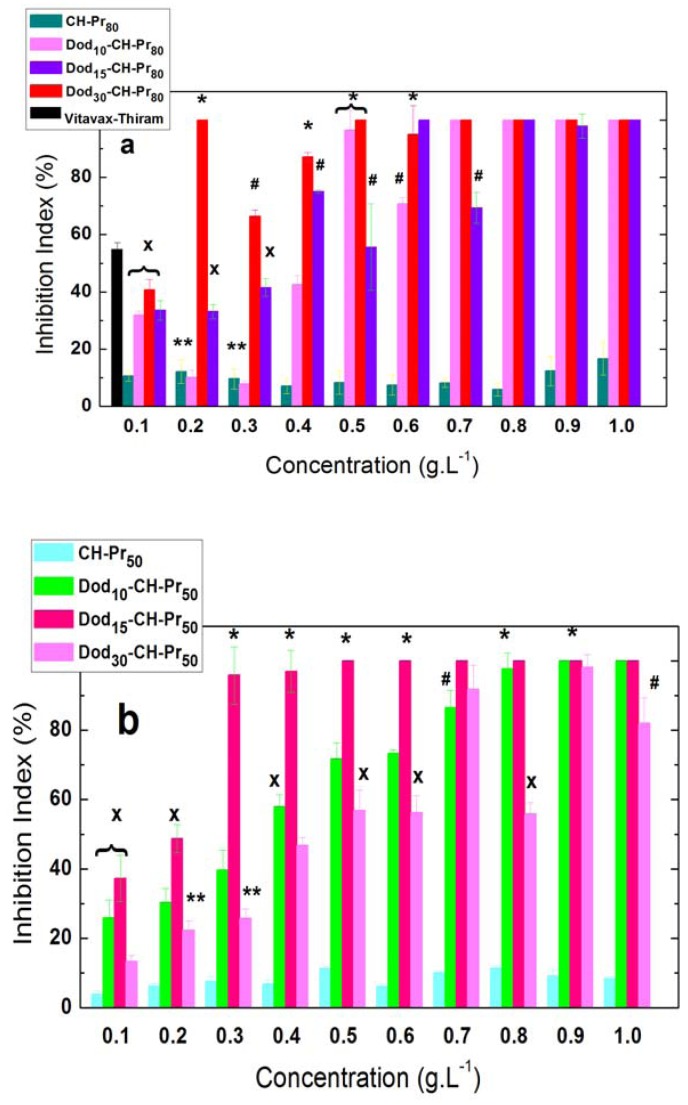
Effect of the hydrophobic modification of the quaternary derivatives of chitosan on the mycelial fungal growth of (**a**) *A. Flavus* and (**b**) *A. parasiticus*. Black bar corresponds to Vitavax^®^-Thiram at 0.1 g.L^−1^ of the crude product. Vertical bars within the same concentration with different symbols are significantly different according to Kruskal-Wallis test (*p* < 0.05).

The less substituted amphiphilic derivatives of both series, Dod_10_-CH-Pr_80_ and Dod_10_-CH-Pr_50_, which contained 8.3 and 7.1 mol % of attached Dod groups, respectively, exhibited, at 0.5 gL^−1^, inhibition indexes of 96.5% and 71.6% against *A. flavus* and *A. parasiticus* respectively, *i.e.*, almost completely inhibited the radial growth of both fungi (Figures 4a,b]. The antifungal activity of Vitavax^®^ Thiram 200 SC, against *A. flavus*, was included in Figure 4(a) for comparative purposes, only at 0.1 g/L, once for higher concentrations the inhibition indexes reached 100%. These indexes of inhibition were confirmed with the subsequent derivatives of the two series, Dod_15_-CH-Pr_80_, Dod_15_-CH-Pr_50_, whose DS_2_ were respectively 14.1 and 9.6%, however Dod_15_-CH-Pr_80_ exhibited similar inhibition efficiency to Dod_10_-CH-Pr_80_. The most substituted derivative Dod_30_-CH-Pr_80_ exhibited the highest activity against *A. flavus*, for instance, at 0.4–0.5 g.L^−1^ these derivatives inhibited around 90–100% of the fungal growth (Figure 4a), while Dod_15_-CH-Pr_80_ reached indexes around 60–70%. On contrary, Dod_15_-CH-Pr_50_ inhibited more efficiently the growth of *A. parasiticus* than the other two polymers of this series, Dod_10_-CH-Pr_50_ and Dod_30_-CH-Pr_50_. However, at 0.9 g/L all the three amphiphilic derivatives prepared from CH-Pr_50_ completely inhibited the fungal growth, as observed with the amphiphilic derivatives prepared from CH-Pr_80_.

Overall, the antifungal activity increases by attaching Dod groups to the polymer chain and the derivative Dod_30_-CH-Pr_80_ substituted with about 30% of Dod groups exhibited a superior performance against *A. flavus*. On the other hand the derivative Dod_15_-CH-Pr_50_, substituted with about 10% of Dod groups was the more efficient against *A. parasiticus.* The effect of different hydrophobic groups attached to chitosan on the antimicrobial activity against other microorganisms, such as *Botrytis cinerea* and *Pyricularia grisea* [11] and *E. coli.* [12], have been studied and indicate that hydrophobicity may be refined to improve antimicrobial activity. Regarding the pathogens chosen for this study, *i.e.*, *A.flavus* and *A. parasiticus*, although comparisons with literature could not be made, a recent study of chitosan modified with N-vanillyl was used to prepare films, and was also studied for their antimicrobial activity against *Aspergillus flavus*. The results showed that these derivatives were able to reduce the aflatoxins produced by the fungus at undetectable levels [19]. Moreover, the *in vitro* antifungal activities against *A. flavus* and *A. parasiticus* reported in this work are similar to those reported using extracts of *Baccharis glutinosa* and *Ambrosia confertiflora* against these fungi [22].

These results were further confirmed by MIC measurements for the series Dod-CH-Pr_50_ and are shown in Table 2. The synthesized amphiphilic derivatives exhibited increasing efficiencies with the degree of substitution by Dod groups, with MICs in the range of 0.125 to 1.0 g/L. As can be seen from Table 2, in the first 48 hours the inhibition of both fungi, *A. flavus* and *A. parasiticus*, by the three amphiphilic derivatives was reached using a much lower polymer concentration (0.125 g.L^−1^) than that needed for CH (> 4.0 g.L^−1^). Although the inhibition provided by deacetylated chitosan in this work is poor compared to those obtained with the amphiphilic derivatives, similar values have been reported for different fungi. For instance, fungistatic activity of chitosan against *A. parasiticus* varied from 6.71 to 10.66 g L^−1^, as evaluated by measuring 50% of colony radial extension growth [19]. 

Rabea *et al.*, reported an EC_50_ (the concentration causing a 50% reduction in radial hyphal growth) of 5.31 g.L^−1^ for chitosan against *Botrytis cinerea*, and the inhibition of chitosan against *Pyricularia grisea* at 5.0 g/L was only 5% [11]. Therefore, the values reported here are within the range reported in literature, even though the chitosan activity being always dependent on molecular weight, degree of acetylation and types of fungi [23]. For a longer incubation time (72 h), higher concentrations of the three amphiphilic derivatives were needed to completely inhibit the growth of the fungi, for instance, 1.0 g.L^−1^ for *A. flavus* and 0.25–0.50 g.L^−1^ for *A. parasiticus*. This behavior may be attributed to the adaptation of the fungus to its new environment, and the same pattern was observed earlier for the cationic derivatives of chitosan without Dod groups [15]. 

The low antifungal activity of deacetylated chitosan against some pathogens may be attributed to the fact that these fungi contains chitin as component of its cell wall [24], therefore a fungistatic mechanism could be postulated based on the interaction of cationic groups with negatively charged cell surface membrane [25], preventing nutrients from entering the cell [26,27]. However, for the amphiphilic systems the presence of hydrophobic groups may favor a stronger anchoring/binding of the amphiphilic chains into the cell wall, leading to a more efficient inhibition. This behavior can result in a mechanism of action that leads to the microbial membrane disruption and cannot be disregarded [28,29].

**Table 2:** Minimum Inhibitory Concentrations of chitosan and its amphiphilic derivatives

Derivative	MIC Concentration (g.L^−1^)			
	*Aspergillus flavus*		*Aspergillus parasiticus*	
	48 h	72 h	48 h	72 h
CH	>4.0	>4.0	>4.0	>4.0
Dod_10_CHPr_50_	0.125	1.0	0.125	0.50
Dod_15_CHPr_50_	0.125	1.0	0.125	0.250
Dod_30_CHPr_50_	0.125	1.0	0.125	0.250

## 3. Experimental

### 3.1. Materials

Chitosan [degree of deacetylation (DD) 85%] was used as starting material and purchased from Polymar Co., Fortaleza, Brazil. Potato Dextrose Agar—PDA—purchased from Acumedia Manufacturers, Inc. Lansing, MI, USA. Tween^®^ 80, sodium hydroxide, sodium Acetate, and acetic acid were purchased from Synth^®^ São Paulo, Diadema, Brazil. (3-Bromopropyl)trimethylammonium bromide, deuterium chloride 35% in deuterium oxide and deuterium oxide, were purchased from Sigma Aldrich Chemical Co., São Paulo, Brazil. Vitavax® Thiram 200 SC, a Dupont/Chemtura trademark, was used as received. The formulation contains 20% (w/v) of carboxin (5,6-dihydro-2-methyl-N-phenyl-1,4-oxathiin-3-carboxamide) and 20% (w/v) thiram (tetramethylthiuram disulfide). Spectra/Pore membranes (Spectrum, Compton, CA, USA) were employed for dialysis. All solvents were of reagent grade and used as received. Water was deionized using a Gehaka water purification system.

### 3.2. Instrumentation

^1^H-NMR spectra were recorded on a Bruker ARX-500 500 MHz spectrometer. pH values of the solutions were measured using a Digimed pH-meter.

### 3.3. Synthesis of the Amphiphilic Propyltrimethylammonium-Modified Chitosans

The synthesis was carried out starting from a fully deacetylated chitosan (CH), obtained via deacetylation of a commercial chitosan sample with a nominal degree of deacetylation (DD) of 85%, following a known procedure [16]. The quaternary derivatives were obtained as described earlier using CH [15]. The resulting polymers were purified by dialysis against water for three days and isolated by lyophilization [15,16]. Next, the quaternary derivatives were subsequently modified with Dod groups by a reductive amination reaction with dodecyl aldehyde (Figure 1). The procedure for the hydrophobic modification of the quaternary derivative is described below for the sample containing 83% of quaternary groups and 10% of Dod groups. The degree of substitution was varied by setting the initial molar ratio as described in Table I. Deacetylated chitosan (1.0 g, 6.2 mmol) was dissolved in acetic acid (0.2 mol·L^−1^, 110 mL), next, ethanol (70 mL) was added to obtain a clear solution. Further, dodecyl aldehyde (0.12 g, 0.65 mmol) dissolved in ethanol (10 mL) was then added dropwise under stirring for about 15 min, and stirring was continued for 30 min at room temperature. The pH of the reaction mixture was adjusted to 5.0 by adding aqueous NaOH (1.0 M). Afterwards the reaction mixture was stirred for 1 h at room temperature. Thereafter, a solution of sodium cyanoborohydride (0.55 g, 8.7 mmol) was added under stirring and the reaction mixture was kept under stirring at room temperature for 24 hrs. The mixture was then dialyzed (membrane of MWCO 6–8,000 g/mol), first against water for 2 days, then against aqueous NaOH (0.05 M) for 1 day, and finally against water for 2 days. The product was isolated by lyophilization and characterized by ^1^H-NMR. 

### 3.4. Characterization

#### 3.4.1. FTIR Analysis

FTIR spectra were recorded on a Perkin Elmer Spectrum 100 Fourier Transform infrared spectrometer; all samples were previously dried at 60 °C overnight under vacuum before collecting data.

#### 3.4.2. Determination of the Degrees of Substitution DS1 and DS2 by 1H-NMR Spectroscopy

^1^H-NMR spectra were recorded at 70 °C. The degree of substitution by the propyltrimethyl-ammonium (Pr) group (DS_1_) was determined as described previously [15] using Equation (1):

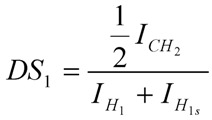
(1)
where I_CH2_ is the area of the signal at δ 2.81 ppm, which corresponds to the resonance of the methylene protons CH_2_***CH_2_***CH_2_N^+^(CH_3_)_3_ (Figure 1) and I_H1_ and I_H1s_ corresponds to the areas of the signals due to the anomeric protons of substituted and unsubstituted glucosamine residues, H_1s_ and H_1_. A similar procedure was used to determine the degree of substitution by the Dod groups (DS_2_) using Equation (2) (Figure 1):

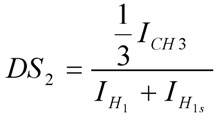
(2)
where, I_CH3_ corresponds to the resonance of methyl protons from dodecy chain, which appear at δ 1.45 ppm. All degrees of substitution are shown in Table 1. 

#### 3.4.3 Viscosity Measurements.

Viscosity measurements were carried out in a water thermostated bath with a capillary calibrated viscosimeter for dilution Cannon-Ubbelohde 9722M-50 (Cannon Instr. Co., State College, PA, USA) at pH 4.5 acetic acid (0.3M)/sodium acetate(0.2 M) buffer. The viscosimeter was immersed in a thermostatic bath at 298.15 ± 0.05 °C and the samples were allowed to equilibrate for 10 min in the bath before measurements. Measurements at each concentration were repeated and the reproducibility was better than ± 0.01 s. The results of the viscosity measurements were expressed as reduced viscosity values calculated from:

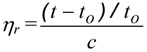
(3)
where *t* is the measured efflux time of the polymer solution, *t_o_* is the efflux time of the pure solvent, and *c* is the polymer concentration (g/L). The mean viscosimetric molecular weight of chitosan and its derivatives were determined by using the Mark-Houwink equation, with constants *a* = 0.796, *K* = 0.079 mL·g^−1^ [30].

### 3.5. Pathogen and Cultures

The microorganisms chosen to test the antifungal activity of chitosan and its derivatives were *Aspergillus flavus* and *Aspergillus parasiticus*. The strains were purchased from the Brazilian Collection of Microorganisms from the Environment and Industry–CBMAI, Campinas–São Paulo, Brazil, and were maintained on potato dextrose agar (PDA) (potato infusion from 200 g/L, 20 g/L dextrose, and 15 g/L agar) in the dark at 25 ± 2 °C.

### 3.6. Antifungal Assays

The antifungal activity of deacetylated was compared to those of the amphiphilic derivatives modified with Dod groups, whose degrees of substitution (DS_2_) were varied from 7.1 to 29%. Stock polymer solutions were prepared in 50 mL of acetic acid 1% (v/v) at pH 5.0 and added to the melted culture medium, which contained 50 mL of 10% potato dextrose broth to obtain polymer concentrations from 0.0 (control plate) to 1.0 g/l, which were then transferred to Petri dishes. After solidification, the mixtures were inoculated with a 2 mm in diameter *Aspergillus flavus* fungus mycelium at the centre of the Petri dishes and were then incubated in an oven for 7 days at 25 ± 2 °C. Inhibition indexes of the fungus by the polymers were determined by the radial growth of the colony with a caliper on the third, fifth and seventh days of cultivation, with the result of the seventh day used for comparative purposes.

The antifungal index was calculated as follows:

Antifungal Index (%) = 1 – (D_a_/D_b_) × 100 (4)

where D_a_ is the diameter of the growth zone in the test plates and D_b_ is the growth zone in the control plate, according to Guo [18]. Each experiment was performed in quadruplicate, and the data were averaged. The Kruskal-Wallis test with Dunn’s multiple comparison were used to evaluate the differences in antifungal index in antifungal tests. Results with *p* < 0.05 were considered statistically significant. 

### 3.7. Minimum Inhibitory Concentrations (MICs)

The MICs were determined using the micro dilution assay in potato dextrose agar (PDA), following the standard method applied to filamentous fungi [31]. The chitosan derivatives were dissolved in aqueous acetic acid at pH 5.0 to obtain stock solutions of 8.0 g·L^−1^. These solutions were subsequently diluted to obtain decreasing polymer concentrations (4.0, 2.0, 1.0, 0.5, 0.25, 0.125; 0.0625, 0.03125, 0,015625, 0.0078 g·L^−1^). Thereafter 100 µL of the polymer solutions were transferred onto 96-well plates and 2 µL of the fungus suspension at a density of 10^6^ conidia/mL were inoculated at each well. The experiments were carried out in triplicate and the MICs were determined by visual inspection of the wells. The lowest concentration that gave complete growth inhibition in all three replicates was recorded as the MIC after 48 and 72 h.

## 4. Conclusions

In this paper the synthesis and characterization of new amphiphilic derivatives of chitosan soluble at neutral pH were reported. The antifungal activities of these derivatives against the fungi *Aspergillus flavus* and *Aspergillus parasiticus* were tested and results demonstrated that the hydrophobic modification of the quaternary derivatives increased antifungal activities. The inhibition indexes for both synthesized series reached complete inhibition at concentrations varying from 0.5 to 1.0 g·L^−1^, which were almost ten times lower than that needed with deacetylated chitosan. The high antifungal activities exhibited by these derivatives can be attributed to the stronger binding of the Dod chains to cell walls and although the mechanism is still unknown, the strengthening of this interaction enhances the antifungal activity. Therefore, the controlled hydrophobic modification and/or variation of the length of the hydrocarbon chain, opens up the possibility to tune the antifungal activity, and depending on the target, to adjust charge surface of the derivatives to boost the activity against other types of fungi and microorganisms. 

## Supplementary Materials

Supplementary materials can be accessed at: http://www.mdpi.com/1420-3049/18/4/4437/s1.

## References

[1] Vinsova J., Vavrikova E. (2011). Chitosan derivatives with antimicrobial, Antitumour and antioxidant activities—A review. Curr. Pharm. Design.

[2] Sajomsang W. (2010). Synthetic methods and applications of chitosan containing pyridylmethyl moiety and its quaternized derivatives: A review. Carbohyd. Polym..

[3] Fu X., Shen Y., Jiang X., Huang D., Yan Y. (2011). Chitosan derivatives with dual-antibacterial functional groups for antimicrobial finishing of cotton fabrics. Carbohyd. Polym..

[4] Rúnarsson Ö.V., Holappa J., Malainer C., Steinsson H., Hjálmarsdóttir M., Nevalainen T., Másson M. (2010). Antibacterial activity of *N*-quaternary chitosan derivatives: Synthesis, Characterization and structure activity relationship (SAR) investigations. Eur. Polym. J..

[5] Kong M., Chen X.G., Xing K., Park H.J. (2010). Antimicrobial properties of chitosan and mode of action: A state of the art review. Int. J. Food Microbiol..

[6] Xu J., Zhao X., Han X., Du Y. (2007). Antifungal activity of oligochitosan against *Phytophthora capsici* and other plant pathogenic fungi *in vitro*. Pest. Biochem. Physiol..

[7] Belalia R., Grelier S., Benaissa M., Coma V. (2008). New bioactive biomaterials based on quaternized chitosan. J. Agric. Food Chem..

[8] Seyfarth F., Schliemann S., Elsner P., Hipler U.-C. (2008). Antifungal effect of high- and low-molecular-weight chitosan hydrochloride, Carboxymethyl chitosan, Chitosan oligosaccharide and *N*-acetyl-d-glucosamine against Candida albicans, Candida krusei *and* Candida glabrata. Int. J. Pharm..

[9] Mohamed N.A., Abd El-Ghany N.A. (2012). Preparation and antimicrobial activity of some carboxymethyl chitosan acyl thiourea derivatives. Int. J. Biol. Macromol..

[10] Li M., Chen X., Liu J., Zhang W., Tang X. (2011). Molecular weight-dependent antifungal activity and action mode of chitosan against *Fulvia fulva* (cooke) ciffrri. J. Appl. Polym. Sci..

[11] Rabea E.I., Badawy M.E.I, Rogge T.M., Stevens C.V., Höfte M., Steurbaut W., Smagghe G. (2005). Insecticidal and fungicidal activity of new synthesized chitosan derivatives. Pest. Manag. Sci..

[12] Sajomsang W., Tantayanon S., Tangpasuthadol V., Daly W.H. (2009). Quaternization of *N*-aryl chitosan derivatives: Synthesis, Characterization, and antibacterial activity. Carbohyd. Res..

[13] Zheng L.-Y., Zhu J.-F. (2003). Study on antimicrobial activity of chitosan with different molecular weights. Carbohyd. Polym..

[14] Amaike1 S., Keller N.P. (2011). Aspergillus flavus. Annu. Rev. Phytopathol..

[15] Pedro R.O., Takaki M., Gorayeb T.C.C., Del Bianchi V.L., Thomeo J.C., Tiera M.J.,  Tiera V.A.O. (2013). Synthesis, Characterization and antifungal activity of quaternary derivatives of chitosan on Aspergillus flavus. Microbiol. Res..

[16] Tiera M.J, Qiu X.P., Bechaouch S., Shi Q., Fernandes J.C., Winnik F.M. (2006). Synthesis and characterization of phosphorylcholine-substituted chitosans soluble in physiological pH conditions. Biomacromolecules.

[17] Xu T., Xin M., Li M., Huang H., Zhou S., Liu J. (2011). Synthesis, Characterization, and antibacterial activity of N,O-quaternary ammonium chitosan. Carbohyd. Res..

[18] Guo Z.Y., Xing R., Liu S., Zhong Z., Ji X., Wang L., Li P. (2007). The influence of the cationic of quaternized chitosan on antifungal activity. Int. J. Food Microbiol..

[19] Cota-Arriola O., Cortez-Rocha M.O., Rosas-Burgos E.C., Burgos-Hernandez A., Lopez-Franco Y.L., Plascencia-Jatomea M. (2011). Antifungal effect of chitosan on the growth of Aspergillus parasiticus and production of aflatoxin B1. Polym. Int..

[20] Zhang B., Wang D.-F., Li H.-Y., Xu Y., Zhang L. (2009). Preparation and properties of chitosan–soybean trypsin inhibitor blend film with anti-*Aspergillus flavus* activity. Ind. Crops Prod..

[21] Jagadish R.S., Divyashree K.N., Viswanath P., Srinivas P., Raj B. (2012). Preparation of *N*-vanillyl chitosan and 4-hydroxybenzyl chitosan and their physico-mechanical, Optical, Barrier, And antimicrobial properties. Carbohyd. Polym..

[22] Rosas-Burgos E.C., Cortez-Rocha M.O., Cinco-Moroyoqui F.J., Robles-Zepeda R.E., López-Cervantes J., Sánchez-Machado D.I., Lares-Villa F. (2009). Antifungal activity *in vitro* of Baccharis glutinosa and Ambrosia confertiflora extracts on *Aspergillus flavus*, *Aspergillus parasiticus* and *Fusarium verticillioides*. World J. Microbiol. Biotechnol..

[23] Sajomsang W., Gonil P., Saesoo S., Ovatlarnporn C. (2012). Antifungal property of quaternized chitosan and its derivatives. Int. J. Biol. Macromol..

[24] Carolyn R., Lee A., Hadwiger A. (1979). The fungicidal effect of chitosan on fungi of varying cell wall composition. Exp. Mycol..

[25] Viera D.B, Carmona-Ribeiro A.M. (2006). Cationic lipids and surfactants as antifungal agents: Mode of action. J. Antimicrob. Chemoth..

[26] Guo Z.Y., Chen R., Xing R., Liu S., Yu H., Wang P. (2006). Novel derivatives of chitosan and their antifungal activities *in vitro*. Carbohyd. Res..

[27] Yang T.C., Chou C.C., Li C.F. (2005). Antibacterial activity of *N*-alkylated disaccharide chitosan derivatives. Int. J. Food Microbiol..

[28] Li P., Poon Y.F., Li W., Zhu H-Y., Yeap S.H., Cao Y., Qi X., Zhou C., Lamrani M., Beuerman R.W. (2011). A polycationic antimicrobial and biocompatible hydrogel with microbe membrane suctioning ability. Nat. Mater..

[29] Schmidtchen A., Pasupuleti M., Morgelin M., Davoudi M., Alenfall J., Chalupka A., Boosting M.M. (2009). Antimicrobial peptides by hydrophobic oligopeptide end tags. J. Biol. Chem..

[30] Roberts G.A.F., Wang W., Martin G.P., Domard A., Muzzarelli R.A.A. (1996). Evaluation of Mark-Houwink viscosimetric constants for chitosan. Advances in Chitin Science.

[31] Clinical And Laboratory Standards Institute (CLSI) (2008). Reference Method for Broth Dilution Antifungal Susceptibility Testing of Filamentous Fungi.

