# Supplementary material for: Hydrophobic Effect of Amphiphilic Derivatives of Chitosan on the Antifungal Activity against Aspergillus flavus and Aspergillus parasiticus
Source: Molecules. 2013 Apr 15;18(4):4437–50. doi: 10.3390/molecules18044437 (PMC6269962; doi:10.3390/molecules18044437)

Supplementary Materials

1. Characterization of Chitosan and its Derivatives: Series Dod_x-CH-Pr_{50}

1.1. Hydrogen Nuclear Magnetic Resonance (^{1}H-NMR)

Figure S1. ^{1}H-NMR spectrum of deacetylated chitosan (CH).

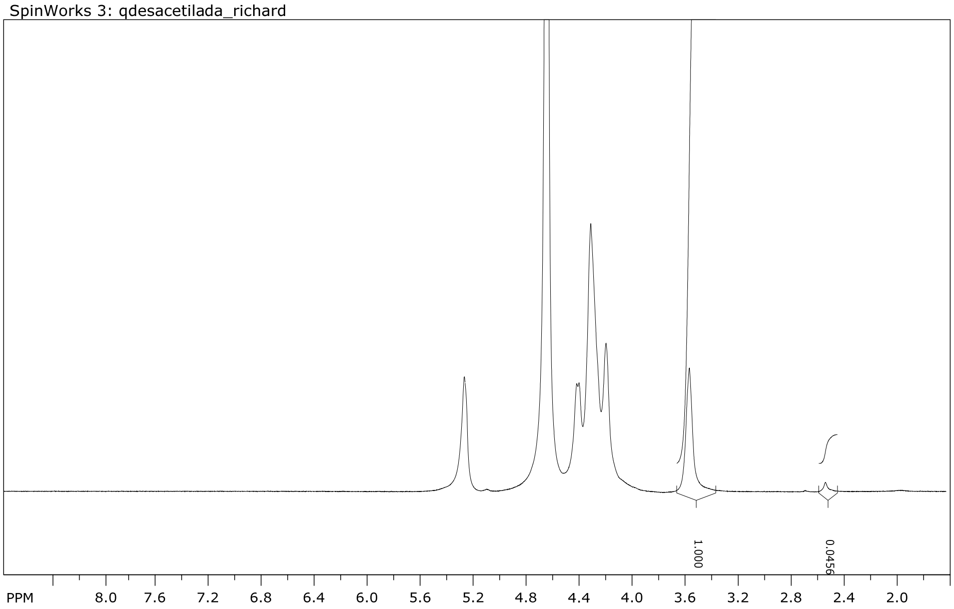

Figure S2. ^{1}H-NMR spectrum of quaternized chitosan - CH-Pr_{50}.

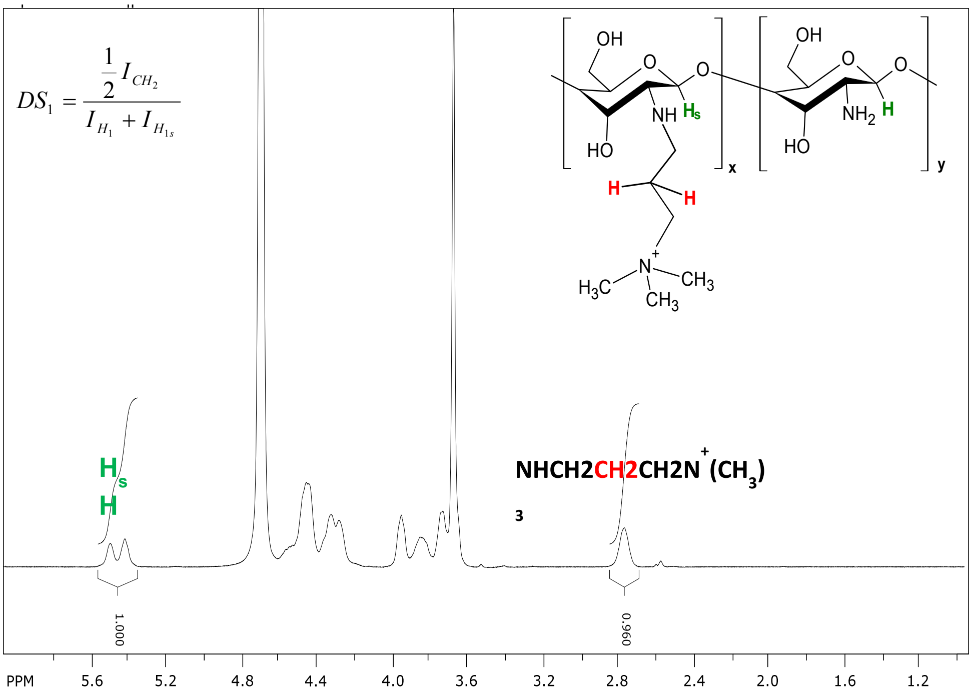

Figure S3. ^{1}H-NMR spectrum of Dod_{10}-CH-Pr_{50}.

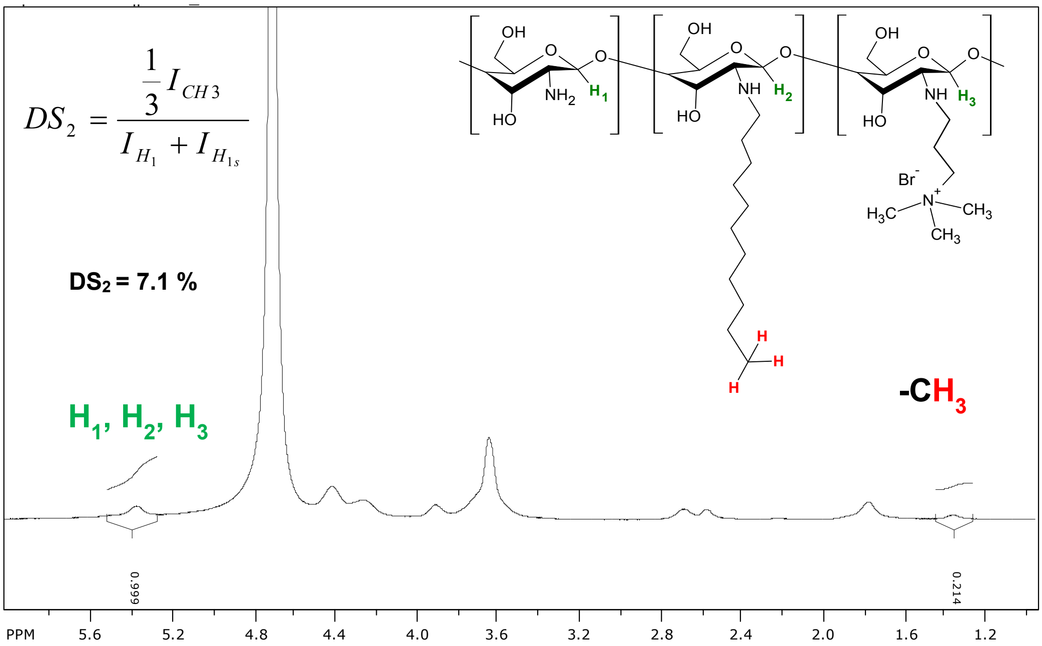

Figure S4. ^{1}H-NMR spectrum of Dod_{15}-CH-Pr_{50}.

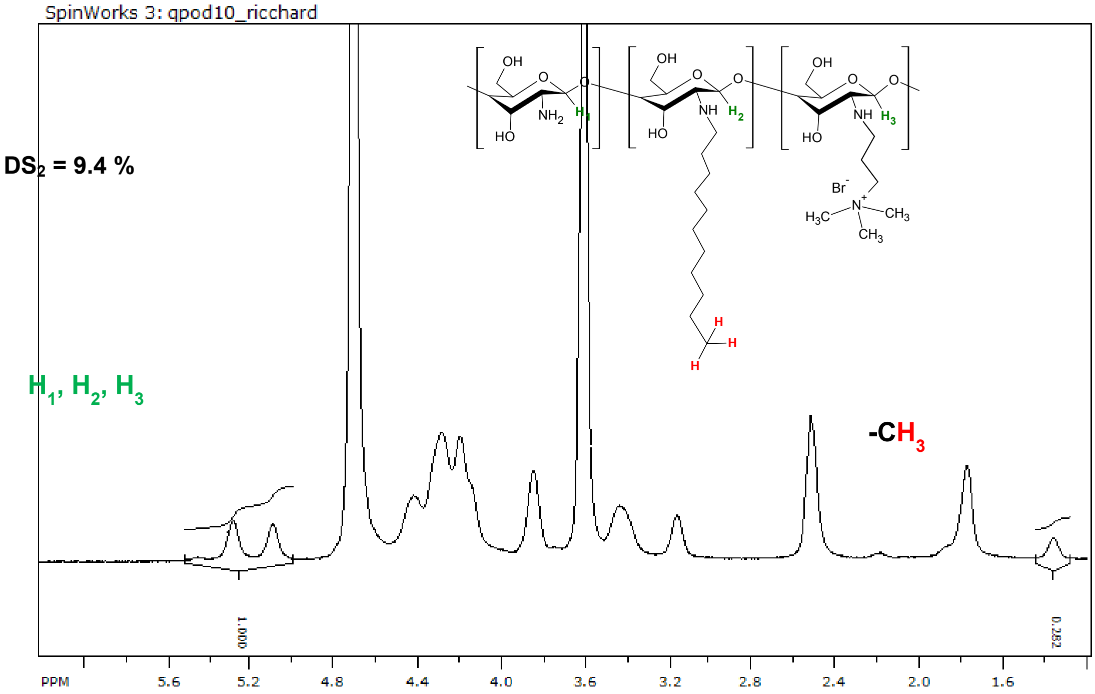

Figure S5. ^{1}H-NMR spectrum of Dod_{30}-CH-Pr_{50}.

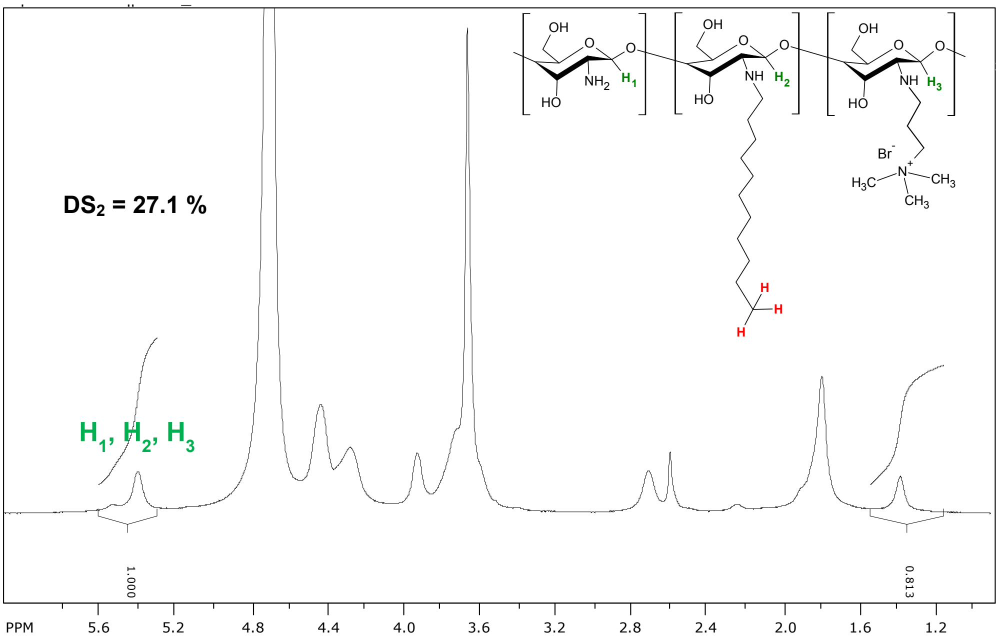

1.2. Viscosity Measurements of CH, CH-Pr_{80} and CH-Pr_{50}

Figure S6. Viscosimetric molecular weight determination of deacetylated chitosan (CH) using Mark Haouwink-Sakurada equation.

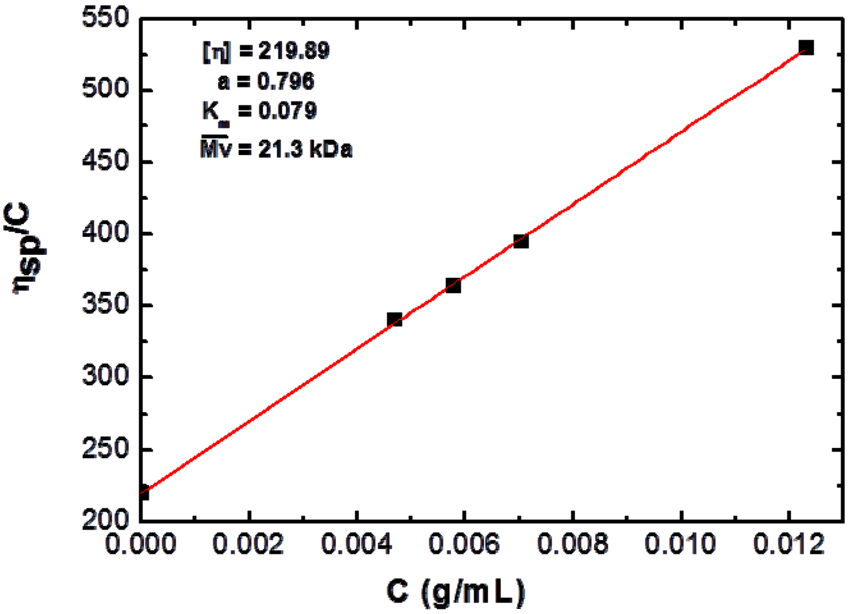

Figure S7. Viscosimetric molecular weight determination of CH-Pr$_{80}$ using Mark Haouwink-Sakurada equation.

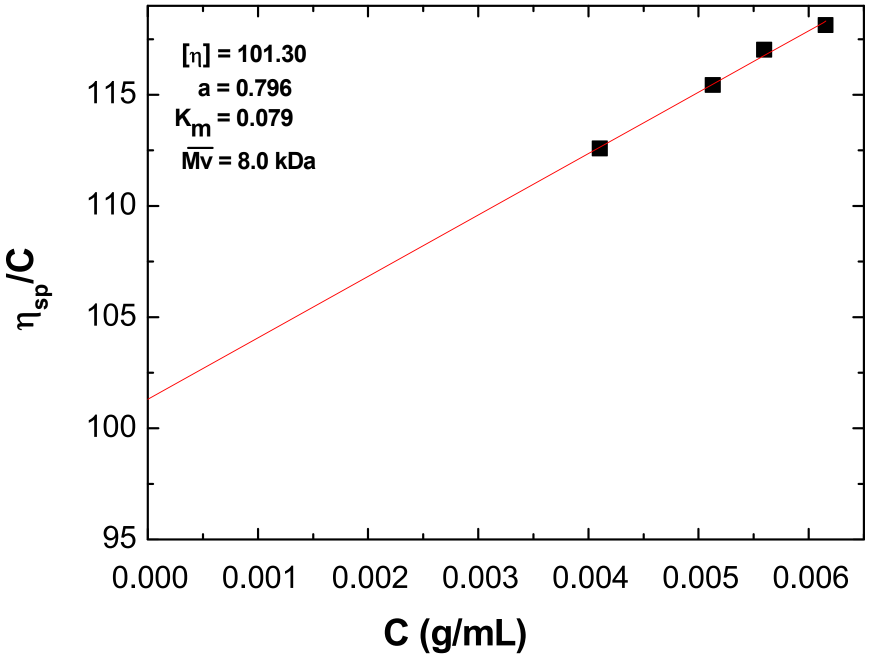

Figure S8. Viscosimetric molecular weight determination of CH-Pr50 using Mark Houwink-Sakurada equation.

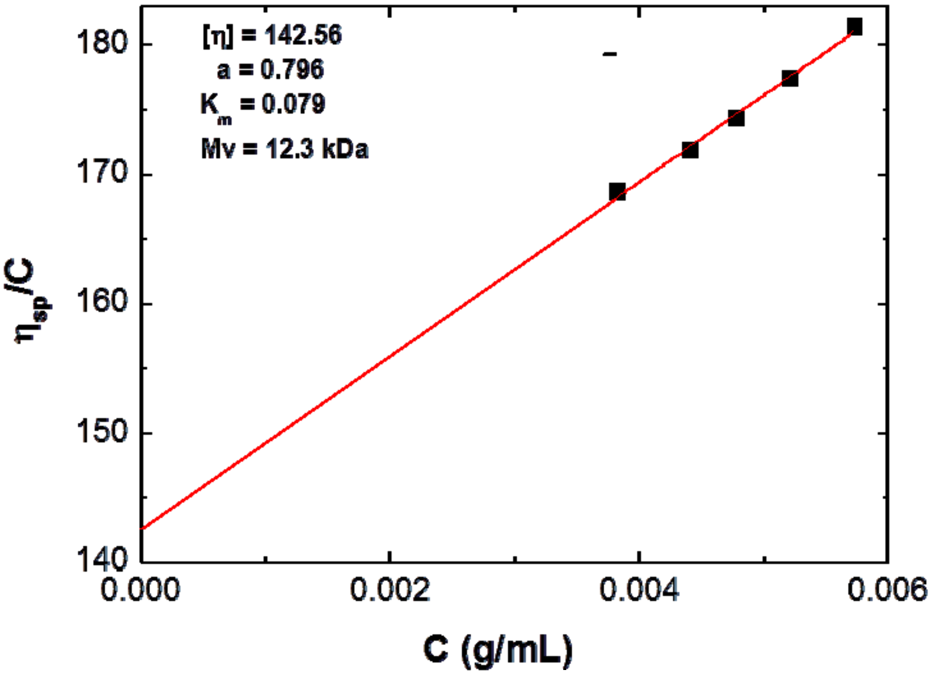

1.3. FTIR Measurements of CH, CH-Pr_{50} and Dod_{30}-CH-Pr_{50}

Figure S9. FTIR spectra of commercial and deacetylated chitosans (CH).

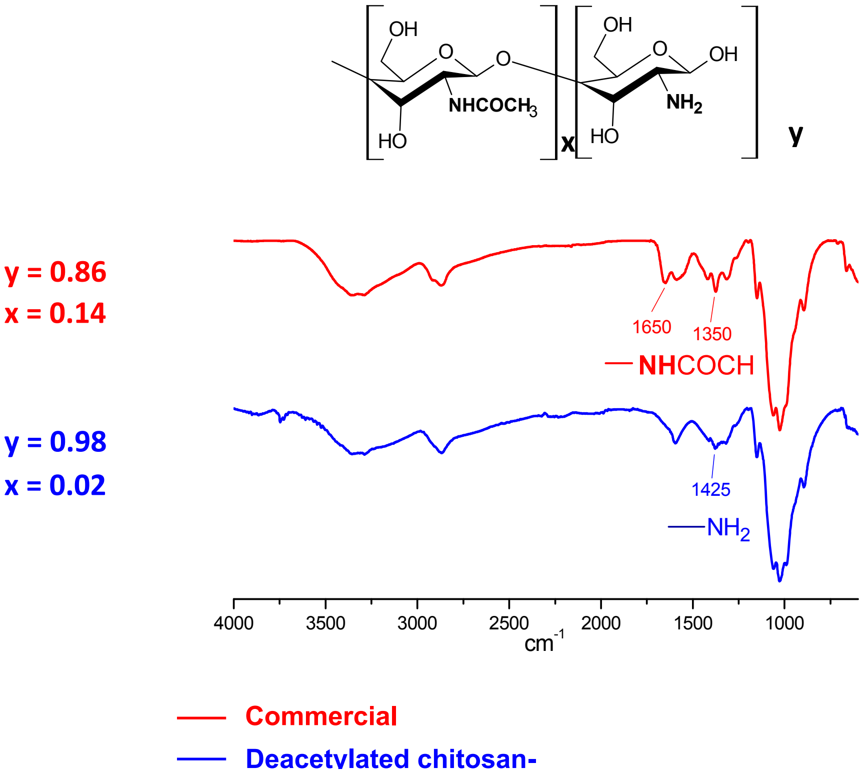

Figure S10. FTIR spectra of deacetylated chitosan and CH-Pr_{50}.

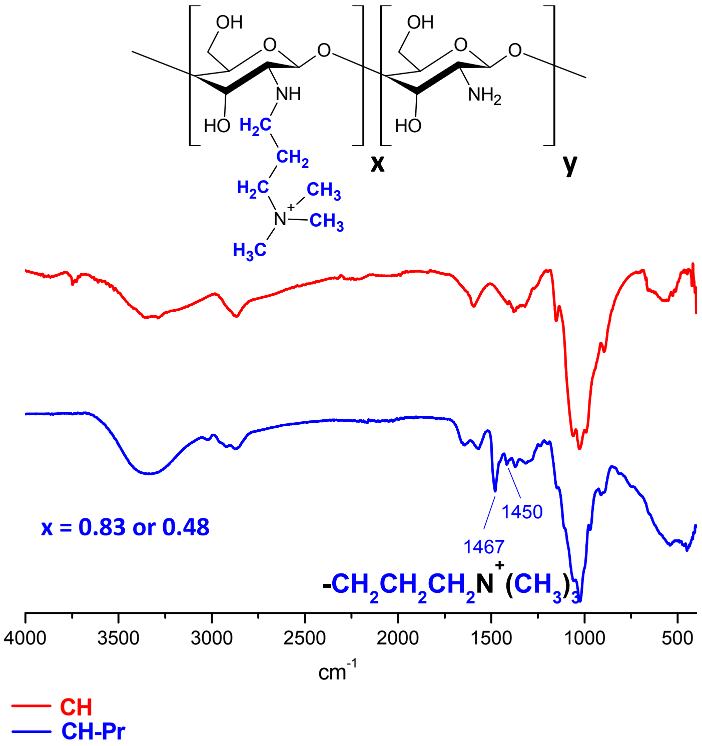

Figure S11. FTIR spectra of deacetylated chitosan, CH-Pr_{50} and Dod_{30}-CH-Pr_{50}.

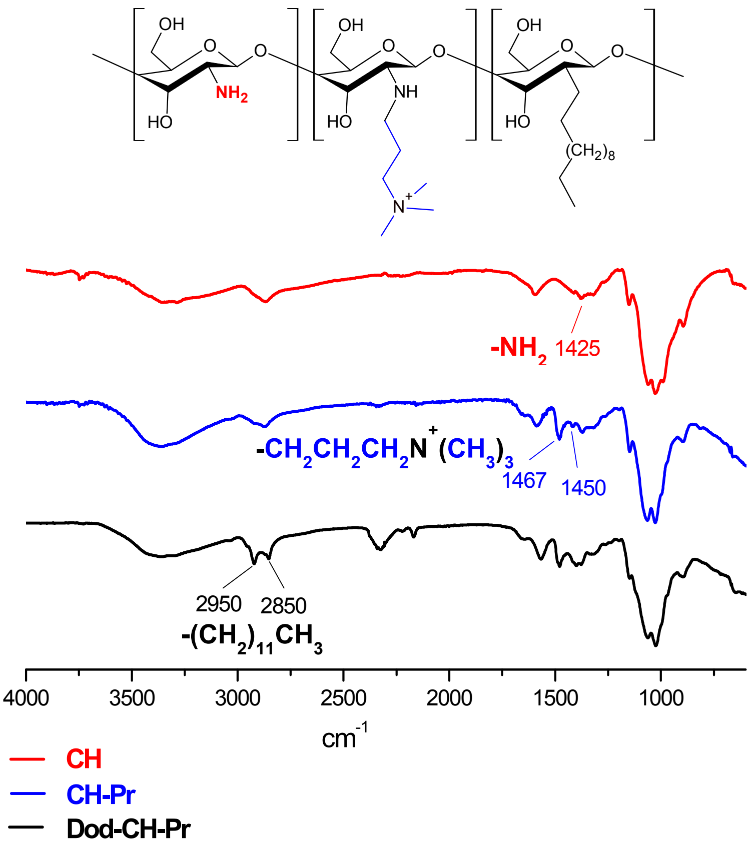

Supplement: Supplementary file 1 [file molecules-18-04437-s001.pdf]
